# 

**Published:** 1868-08-15

**Authors:** 


### *CONTENTS:*

ORIGINAL PAPERS:

Experimental Physiology. By Walter Hay, M.D., Associate Editor, Chicago ..... 515

Notes and Observations in General Practice. By John Forman, M. D., Edinburgh ..... 524

CORRESPONDENCE:

Letter from A. J. Miller, M. D ..... 532

FOREIGN CORRESPONDENCE:

Letter from Samuel Cole, M. D., Berlin, Prussia ..... 537

Fougera's Cod Liver Oil ..... 542

EDITORIAL:

News and Gossip ..... 544

Periodicals Announced ..... 545

Clippings from Correspondence ..... 546

